# Infrared imaging and spectral-domain optical coherence tomography findings correlate with microperimetry in acute macular neuroretinopathy: a case report

**DOI:** 10.1186/1752-1947-5-536

**Published:** 2011-10-31

**Authors:** Sandeep Grover, Vikram S Brar, Ravi K Murthy, Kakarla V Chalam

**Affiliations:** 1 Department of Ophthalmology, University of Florida College of Medicine, Jacksonville, Florida, USA

## Abstract

**Introduction:**

Spectral-domain optical coherence tomography findings in a patient with acute macular neuroretinopathy, and correlation with functional defects on microperimetry, are presented.

**Case presentation:**

A 25-year old Caucasian woman presented with bitemporal field defects following an upper respiratory tract infection. Her visual acuity was 20/20 in both eyes and a dilated fundus examination revealed bilateral hyperpigmentary changes in the papillomacular bundle. Our patient underwent further evaluation with spectral-domain optical coherence tomography, infrared and fundus autofluorescence imaging. Functional changes were assessed by microperimetry. Infrared imaging showed the classic wedge-shaped defects and spectral-domain optical coherence tomography exhibited changes at the inner segment-outer segment junction, with a thickened outer plexiform layer overlying these areas. Fluorescein and indocyanine green angiography did not demonstrate any perfusion defects or any other abnormality. Microperimetry demonstrated focal elevation in threshold correlating with the wedge-shaped defects in both eyes.

**Conclusion:**

Spectral-domain optical coherence tomography findings provide new evidence of the involvement of the outer plexiform layer of the retina in acute macular neuroretinopathy.

## Introduction

Acute macular neuroretinopathy (AMNR) is a rare condition characterized by wedge-shaped lesions pointing towards the foveal center, resulting in bilateral or unilateral scotomas, typically with preserved central visual acuities [1,2]. The association of this condition with oral contraceptive (OCP) use and intravenous sympathomimetic administration suggests a vascular etiology, although angiography has consistently failed to demonstrate a perfusion defect [2]. Findings on time domain optical coherence tomography (OCT) indicate that the pathology is located in the outer retina [3].

We present findings of infrared (IR) imaging and spectral-domain OCT (SD-OCT; Spectralis, Heidelberg, Germany) and correlate these with retinal function by microperimetry. The findings demonstrate outer plexiform layer (OPL) thickening in this case of AMNR.

## Case presentation

A 25 year-old Caucasian woman presented with a four-day history of acute onset of blurred vision in both eyes. She reported a viral upper respiratory tract infection for seven to 10 days, for which she had taken two Excedrin^® ^Migraine (acetaminophen 250 mg, aspirin 250 mg and caffeine 65 mg) tablets. She used Midrin (acetaminophen 325 mg, dichloralphenazone 100 mg, isometheptene mucate 65 mg) as needed for her headaches concurrently. Additionally, she smoked half-pack cigarettes and consumed four to five 12-ounce cans of a caffeinated drink, Mountain Dew (caffeine 54 mg/can) per day.

**Figure 1:**
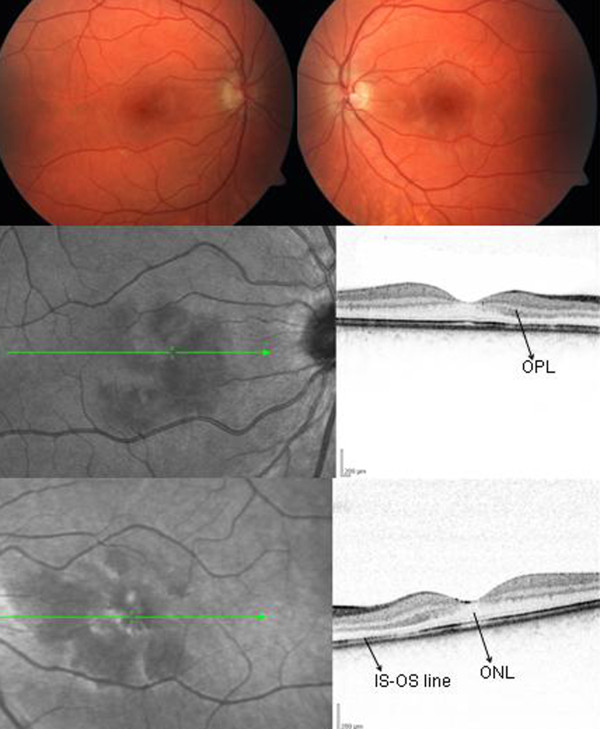
**Color fundus photographs**. (Top panel) Images of the right and left eye reveal subtle irregularities of the internal limiting membrane reflex and pigmentary changes. IR imaging with corresponding Spectralis OCT cross- sectional image of the right (Middle panel) and left (Bottom panel) eye reveals classic wedge-shaped lesions. Spectralis OCT demonstrates thickening of the OPL with underlying thinning of the outer nuclear layer. The IS-OS line is affected in both eyes.

Her uncorrected Snellen visual acuity was 20/20 in both eyes and Amsler testing revealed bitemporal paracentral scotomas. She correctly identified 10 and nine out of 14 Ishihara color plates, in her right and left eye, respectively. No afferent pupillary defect was noted and the anterior segment was unremarkable. Fundus examination revealed bilateral hyperpigmentary changes in the papillomacular bundle (Figure 1A). Fundus autofluorescence revealed a normal autofluorescence pattern. IR imaging disclosed classic wedge-shaped lesions with their apices oriented towards the fovea. SD-OCT exhibited changes at the inner segment-outer segment (IS-OS) junction, with a thickened OPL overlying these areas (Figures 1B, C). Humphrey visual field (HVF) 30-2 demonstrated bilateral paracentral scotomas. Fluorescein and indocyanine green angiography did not demonstrate any perfusion defects or any other abnormality.

**Figure 2:**
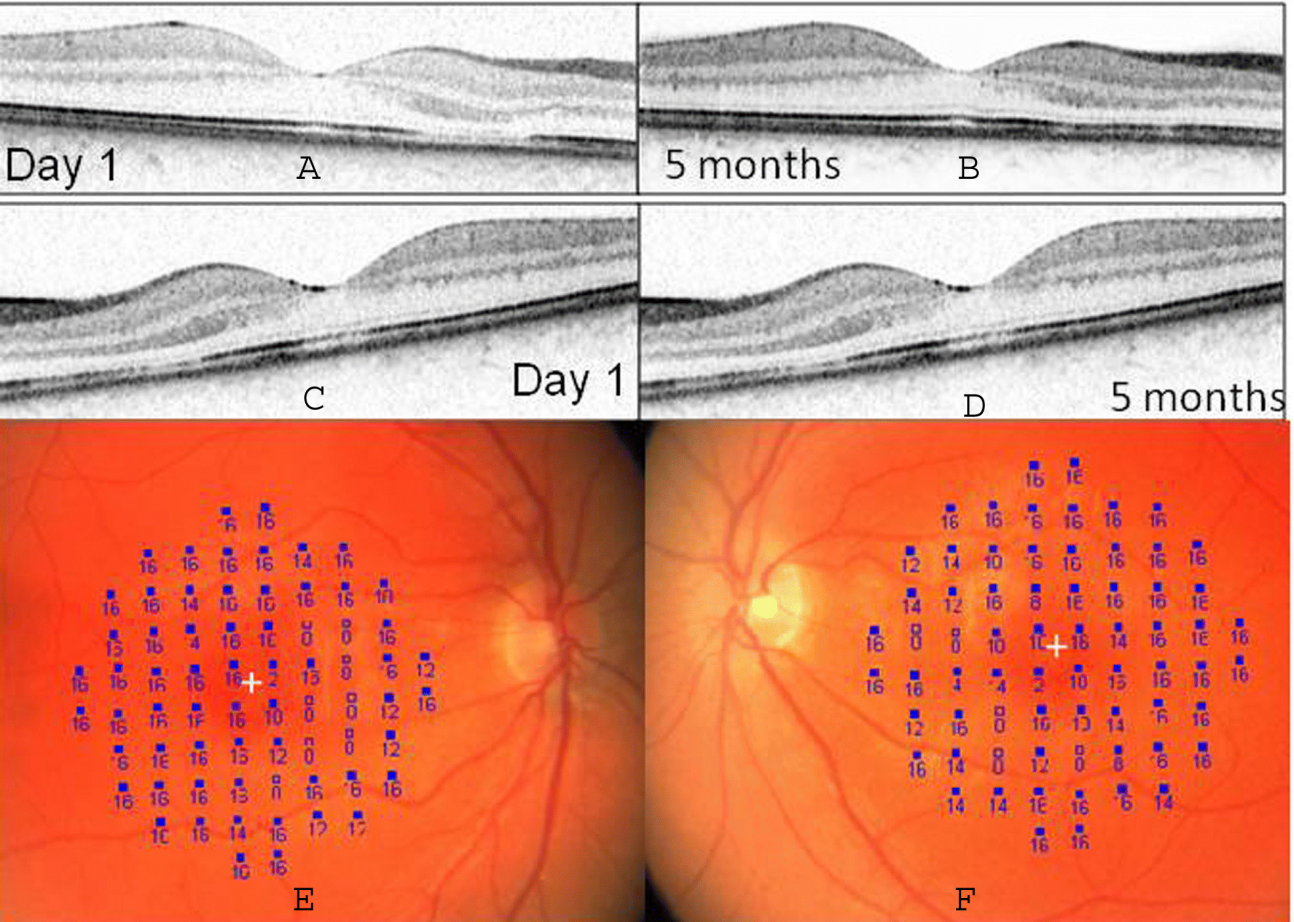
**Sequential Spectralis OCT images**. The right eye at **(A) **the time of initial presentation and **(B) **five months later showing small improvement in the IS-OS junction. The left eye at **(C) **the time of presentation and **(D) **five months later did not show any improvement. **(E and F) **Microperimetry demonstrated elevation in threshold in the area of the lesion in both eyes (right eye, E and left eye, F).

Five months after initial presentation, her color vision improved to 14 of 14 Ishihara color plates correctly identified in each eye. Repeat HVF testing demonstrated interval improvement in the scotomas, more in her right eye than left. Similarly, SD-OCT showed a corresponding small improvement at the IS-OS junction in her right eye (Figure 2A and 2B) and no change in her left eye (Figure 2C and 2D). Microperimetry using an MP-1 (Nidek, Japan) demonstrated focal elevation in threshold correlating with the wedge-shaped defects in both her eyes (Figure 2E and 2F).

## Conclusion

AMNR remains an elusive condition in regards to the etiology of retinal lesions. Eighty-three percent of cases affect younger women, nearly half of whom report an associated viral illness [1]. Other reported associations include OCP use and the intravenous administration of epinephrine (ranging from 0.5 mL in a 1:1000 solution to 10 mg) and ephedrine (25 mg) [2]. Our patient was not taking OCP and reports oral decongestant use only. She did report consuming caffeine of up to 270 mg per day, which is far less than that reported in cases of "caffeine-doughnut maculopathy" [4].

In our patient, the characteristic lesions were not seen on fundus examination, but were clearly evident on IR imaging. Fundus autofluorescence did not demonstrate an abnormal autofluorescence pattern, indicating that the retinal pigment epithelium was not affected. Two recent reports demonstrated localization of the retinal lesions in AMNR to the photoreceptor IS/OS junction, using ultra-high resolution OCT [5,6]. SD-OCT findings in our patient confirmed these findings but additionally, we noted focal thickening of the OPL overlying these lesions. Microperimetry demonstrated the presence of elevated threshold corresponding to the area of OPL thickening. The presence of OPL involvement confirms the disease process to the outer retina.

## Consent

Written informed consent was obtained from the patient for publication of this case report and any accompanying images. A copy of the written consent is available for review by the Editor-in-Chief of this journal.

## Competing interests

The authors declare that they have no competing interests.

## Authors' contributions

VB and SG were responsible for the clinical follow-up of our patient. RM, SG and KC were responsible for editing and critical review of the manuscript. All authors have read and approved the final manuscript.
